# Indacaterol/glycopyrronium is cost-effective compared to salmeterol/fluticasone in COPD: FLAME-based modelling in a Swedish population

**DOI:** 10.1186/s12931-017-0688-5

**Published:** 2017-12-11

**Authors:** Leif Bjermer, Job F. M. van Boven, Madlaina Costa-Scharplatz, Dorothy L. Keininger, Florian S. Gutzwiller, Karin Lisspers, Ronan Mahon, Petter Olsson, Nicolas Roche

**Affiliations:** 1 Department of Respiratory medicine & Allergology, Skane University Hospital, Lund University, Lund, Sweden; 2 Department of General Practice, Groningen Research Institute for Asthma and COPD (GRIAC), University Medical Center Groningen, University of Groningen, Groningen, The Netherlands; 3 Unit of Pharmacoepidemiology & Pharmacoeconomics, Department of Pharmacy, University of Groningen, Groningen, The Netherlands; 4 Novartis Sverige AB, Täby, Sweden; 5 Novartis Pharma AG, Basel, Switzerland; 6 Department of Public Health and Caring Sciences, Family Medicine and Preventive Medicine, Uppsala University, Uppsala, Sweden; 7 Novartis Ireland Limited, Dublin, Ireland; 8 Respiratory and Intensive Care Medicine, Cochin Hospital (AP-HP) and University Paris Descartes, Paris, France

**Keywords:** Chronic obstructive pulmonary disease, Indacaterol/glycopyrronium, Cost-effective, Exacerbation

## Abstract

**Background:**

This study assessed the cost-effectiveness of indacaterol/glycopyrronium (IND/GLY) versus salmeterol/fluticasone (SFC) in chronic obstructive pulmonary disease (COPD) patients with moderate to very severe airflow limitation and ≥1 exacerbation in the preceding year.

**Methods:**

A previously published and validated patient-level simulation model was adapted using clinical data from the FLAME trial and real-world cost data from the ARCTIC study. Costs (total monetary costs comprising drug, maintenance, exacerbation, and pneumonia costs) and health outcomes (life-years (LYs), quality-adjusted life-years (QALYs)) were projected over various time horizons (1, 5, 10 years, and lifetime) from the Swedish payer’s perspective and were discounted at 3% annually. Uncertainty in model input values was studied through one-way and probabilistic sensitivity analyses. Subgroup analyses were also performed.

**Results:**

IND/GLY was associated with lower costs and better outcomes compared with SFC over all the analysed time horizons. Use of IND/GLY resulted in additional 0.192 LYs and 0.134 QALYs with cost savings of €1211 compared with SFC over lifetime. The net monetary benefit (NMB) was estimated to be €8560 based on a willingness-to-pay threshold of €55,000/QALY. The NMB was higher in the following subgroups: severe (GOLD 3), high risk and more symptoms (GOLD D), females, and current smokers.

**Conclusion:**

IND/GLY is a cost-effective treatment compared with SFC in COPD patients with mMRC dyspnea grade ≥ 2, moderate to very severe airflow limitation, and ≥1 exacerbation in the preceding year.

**Electronic supplementary material:**

The online version of this article (10.1186/s12931-017-0688-5) contains supplementary material, which is available to authorized users.

## Summary

Indacaterol/glycopyrronium is more effective and cost saving vs salmeterol/fluticasone in Swedish COPD patients with a history of exacerbations.

## Background

Chronic obstructive pulmonary disease (COPD) is a preventable and treatable disease characterised by persistent respiratory symptoms and airflow limitation and is a major cause of morbidity and mortality throughout the world [1–4]. In the European Union, the total direct and indirect cost for COPD amounts to nearly €48 billion [5]. In Sweden, the prevalence of COPD was reported to be 16.2%; 6.8% men and 6.6% women aged ≥40 years had spirometric stage II and higher COPD [6]. The societal costs of COPD in Sweden are high, with total annual costs estimated to be €1.5 bn (SEK 13.9 bn) in 2010, where 35% accounted for direct costs and 65% for indirect costs [7]. A survey reported that subjects with moderate and severe/very severe COPD accounted for 37% and 3% of the studied population (subjects with COPD aged 39–84 years living in northern Sweden), but contributed to 80% of the total COPD costs in Sweden (66% and 14%, respectively) [7].

According to the international Global Initiative for Chronic Obstructive Lung Disease (GOLD) report, COPD treatment aims to reduce exacerbations and improve quality of life [1]. For that purpose, besides non-pharmacologic treatments, several medications are available including bronchodilators and inhaled corticosteroids. The 2017 GOLD report recommends the first line use of dual bronchodilators, such as combination of the long-acting β2-adrenergic agonist (LABA) indacaterol and the long-acting muscarinic antagonist (LAMA) glycopyrronium (IND/GLY), in the treatment of symptomatic COPD patients, regardless of their exacerbation risk [1]. In contrast, the use of inhaled corticosteroid (ICS)-containing combination therapies, such as salmeterol/fluticasone (SFC) may only be a first choice therapy in COPD patients with features of asthma [1]. Key evidence for this recent GOLD strategy comes from the FLAME trial which demonstrated the superiority of IND/GLY in significantly reducing the rate of moderate or severe COPD exacerbations by 17% vs salmeterol/fluticasone (SFC) and increasing time-to-first moderate or severe exacerbation in patients with dyspnoea modified Medical Research Council (mMRC) scale grade ≥ 2 and a documented history of ≥1 moderate or severe COPD exacerbations during the previous year [8]. In addition, the incidence of pneumonia was significantly lower in patients on IND/GLY than in those on SFC (3.2% vs. 4.8%, *p* = 0.02). This, along with the results demonstrated in the ILLUMINATE and LANTERN trial, indicates that IND/GLY addresses needs for both exacerbating and non-exacerbating patients, with a lower risk of pneumonia (the clinical significance of reduced incidence of pneumonia remains to be elucidated) than ICS-containing regimens [9, 10].

The Swedish health care system is financed by a social insurance that provides all citizens with subsidised healthcare through the government. For prescribed drugs fees to the user are capped at 2200 Swedish Krona (SEK) or around €230 per annum. The dual bronchodilator IND/GLY is approved and reimbursed in Sweden for the maintenance treatment for COPD patients remaining symptomatic on long-acting bronchodilator monotherapy [11].

Because COPD carries a significant health and economic burden, available therapies should be critically evaluated for their costs and benefits when making treatment decisions. Indeed, two previously conducted cost-effectiveness analyses (CEAs) have shown favourable cost-effectiveness of IND/GLY compared with SFC in patients with moderate-to-severe COPD and a history of one or no exacerbation in the previous year [12, 13]. Given changing drug treatment costs and the growing role of LABA/LAMAs in the GOLD 2017 strategy new economic evaluations are needed.

This analysis therefore aimed to determine the health economic impact of IND/GLY and SFC as competing treatment options in COPD patients with moderate to very severe airflow limitation and a history of ≥1 exacerbation in the preceding year.

## Methods

### Study design

A previously published and validated microsimulation model [14], was employed to assess the cost-effectiveness (a type of economic evaluation that compares relative costs and outcomes of two or more treatments) of IND/GLY compared with SFC, and was adapted for the present analysis by incorporating clinical data from the FLAME study and real-world Swedish cost data.

### Perspective

The analysis was conducted from a Swedish payer’s perspective. Only direct costs were considered for the analysis.

### Patient population

The FLAME study was a 52-week, phase IIIB, multi-centre, randomised, double-blind, double-dummy, parallel-group, non-inferiority trial that included adults aged ≥40 years, with a clinical diagnosis of COPD, with a mMRC score ≥ 2, a post-bronchodilator forced expiratory volume in 1 s (FEV_1_) of ≥25% predicted to <60% predicted, and a post-bronchodilator ratio of FEV_1_ to forced vital capacity (FVC) of <0.70 [8]. In addition, patients had a documented history of ≥1 COPD exacerbation during the previous year for which they had received treatment with systemic glucocorticoids, antibiotic agents, or both. Table 1 represents the baseline characteristics of the FLAME study population.

**Table 1:** FLAME patient population baseline characteristics

Baseline characteristics	Values
Age at baseline, mean (SD), years	64.6 (7.8)
Height, mean (SD), cm	169 (8.7)
Weight, mean (SD), kg	73.9 (17.1)
BMI, mean (SD), kg/m^2^	25.9 (5.2)
Proportion males, n (%)	2557 (76.1)
Severity of COPD	
GOLD 1^a^, *n* (%)	0 (0.0)
GOLD 2^a^, *n* (%)	1123 (33.7)
GOLD 3^a^, *n* (%)	1954 (58.6)
GOLD 4^a^, *n* (%)	257 (7.7)
Group A^b^, *n* (%)	2 (0.1)
Group B^b^, *n* (%)	822 (24.4)
Group C^b^, *n* (%)	3 (0.1)
Group D^b^, *n* (%)	2514 (74.8)
Number of COPD exacerbations in the previous year	1.19
Current smokers, *n* (%)	1333 (39.6)

*BMI* body mass index, *COPD* chronic obstructive pulmonary disease, *GOLD* global initiative for chronic obstructive lung disease, *SD* standard deviation

^a^Severity of airflow limitation based on 2011–2014 GOLD criteria; ^b^Based on 2015 GOLD staging system

### Model structure

**Fig. 1:**
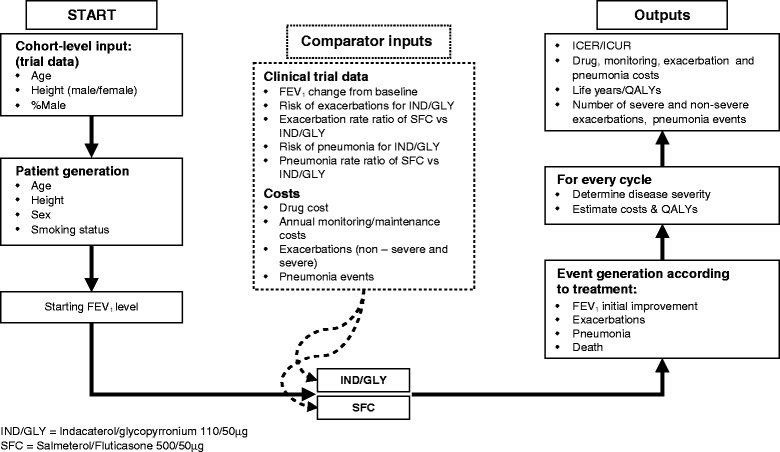
Model structure. Figure notes: BMI: body mass index; FEV_1_: forced expiratory volume in 1 s; ICER: incremental cost-effectiveness ratio; ICUR: incremental cost-utility ratio; IND/GLY: indacaterol/glycopyrronium; NNT: number needed to treat; QALY: quality-adjusted life-year; SFC: salmeterol/fluticasone

A patient-level simulation model was chosen over a cohort model because it is better suited to simultaneously account for different aspects of a patient’s profile such as smoking status, GOLD FEV_1_ status and exacerbation history, and it better reflects the heterogeneous disease progression in COPD patients [15]. Figure 1 shows the structure of the model. In-depth model mechanics and validation have been previously published and the model has been used in an earlier assessment of dual bronchodilation by IND/GLY [12–14]. This model was adapted to the Swedish setting using exacerbation and maintenance costs from the ARCTIC study, a large, real-world retrospective Swedish cohort study of 18,586 eligible primary care COPD patients [16–18]. Other inputs such as costs data, utilities and mortality data were derived from publicly available sources to compare IND/GLY with SFC.

#### Disease progression

A simulated cohort of 100,000 patients was assigned baseline characteristics derived from the FLAME trial. The model then generated patients based on the mean values and variance-covariance matrices derived from patient-level trial data. In the simulation, a generated patient moved through the model in cycles of 6 months, experiencing disease progression and clinical events based on their characteristics and pre-defined probabilities of experiencing events until death or the end of the time horizon. These clinical events included FEV_1_ decline, exacerbations and pneumonia events. A disease severity level was estimated at each cycle. The patient’s disease status was represented by their percent predicted FEV_1_ score, which was generated for each patient according to their baseline characteristics. Treatment-specific FEV_1_ improvements as reported in the FLAME trial were added to each treatment group. Increase over baseline FEV_1_ was assumed to be maintained over time. The patient’s FEV_1_ declined over time at a rate described for the general population studied by Falaschetti et al. [19]. As FEV_1_ declines, patients move into GOLD airflow limitation stages of increasing severity.

#### Exacerbations and pneumonia

An annualised rate of moderate and severe exacerbations adjusted for cycle length was applied, with a probability that a patient experienced either a moderate or severe exacerbation at each cycle (proportion calculation based on number of exacerbations/ total exacerbations as reported in the FLAME study). Though the FLAME study assessed all exacerbations including mild exacerbations, the present analyses only focused on moderate and severe exacerbations (see Additional file 1 for definitions). Pneumonia incidence rates and costs were calculated considering inclusion of an ICS comparator and the established evidence of risk of pneumonia with ICS use [20].

#### Time horizon

Cumulative costs and health outcomes for both IND/GLY and SFC were estimated using a lifetime horizon (considered to be 78 years when ~100% patients are dead) according to Swedish guidelines [21]. Treatment effects were assumed to be constant over the lifetime horizon. To better inform healthcare policies on the short-term, different time horizons (1, 5 and 10 years) were used in the scenario analyses.

### Model input parameters

#### Efficacy

The efficacy inputs used in the analysis were derived from the FLAME trial for the modified intention-to-treat population (Table 2). The FLAME trial demonstrated superior efficacy of IND/GLY over SFC in reducing the annual rate of moderate and severe exacerbations, improvement in trough FEV_1_, health-related quality-of-life, and decrease in the use of rescue medication [8]. Definitions of exacerbation and pneumonia are provided in the Additional file 1.

**Table 2:** Model inputs

Parameter	Mean	Variance	Source
Clinical efficacy			
Annual rate of moderate and severe exacerbations IND/GLY	0.98	CI: 0.88—1.1	[8]
Annual rate of moderate and severe exacerbations SFC	1.19	CI: 1.07—1.32	[8]
LS mean improvement in pre-dose trough FEV_1_ from baseline in litres at 52 weeks IND/GLY	0.015	CI: 0.000—0.030	[8]
LS mean improvement in pre-dose trough FEV_1_ from baseline in litres at 52 weeks SFC	−0.048	CI: −0.063—(−0.033)	[8]
Pneumonia incidence rate IND/GLY	0.035	CI: 0.026—0.044	[8]
Pneumonia incidence rate SFC	0.054	CI: 0.042—0.066	[8]
Costs (€)			
Drug costs (per day) IND/GLY	1.50	CI: 1.32—2.19	[22]
Drug costs (per day) SFC	1.43	CI: 1.25—2.08	[22]
Moderate exacerbation cost per occurrence Moderate airflow limitation	544 (median: 197)	SD: 893	[16]
Moderate exacerbation cost per occurrence Severe airflow limitation	530 (median: 221)	SD: 712	[16]
Moderate exacerbation cost per occurrence Very severe airflow limitation	481 (median: 219)	SD: 705	[16]
Severe exacerbation cost per occurrence Moderate airflow limitation	5168 (median: 3616)	SD: 5282	[16]
Severe exacerbation cost per occurrence Severe airflow limitation	5172 (median: 3959)	SD: 5136	[16]
Severe exacerbation cost per occurrence Very severe airflow limitation	7180 (median: 4584)	SD: 7706	[16]
Annual non-exacerbation related maintenance costs Moderate airflow limitation	5936	–	[16]
Annual non-exacerbation related maintenance costs Severe airflow limitation	5760	–	[16]
Annual non-exacerbation related maintenance costs Very severe airflow limitation	6493	–	[16]
Pneumonia costs	4822	–	[23]

Costs (€) were inflated to the year 2015

*CI* confidence interval, *FEV*_*1*_ forced expiratory volume in 1 s, *GOLD* global initiative for chronic obstructive lung disease, *IND/GLY* indacaterol/glycopyrronium, *LS* least squares, *SD* standard deviation, *SFC* salmeterol/fluticasone

The annualised rates of pneumonia-related events were derived from the incidence of pneumonia reported at 52 weeks and the total number of treatment years in the FLAME study by the following equation (Table 2).

Annualized rate = number of events/person years, where person years = ~N/2.

#### Costs

The cost items considered were drugs for COPD treatment, maintenance/monitoring, exacerbations (moderate and severe) and pneumonia events. Daily drug costs were derived from the Swedish Pharmaceutical Benefits Agency (TLV) [22]. Table 2 shows the drug costs (per day) used in the analysis. Both maintenance and exacerbation costs were sourced from a burden of illness analysis in the ARCTIC study (Table 2) [16]. The cost inputs used and their definitions are provided in the Additional file 1.

Moderate exacerbation costs comprised the following costs during 14 days after the exacerbation occurrence: outpatient visits, nurse visits, physician visits, oral steroids, and antibiotics targeted at respiratory diseases [16]. Moderate exacerbation costs are low and independent of the severity of airflow limitation. Severe exacerbation costs comprised all the components of moderate exacerbation costs and costs of hospital admissions [16]. There may be outpatient costs in patients hospitalized for exacerbations, corresponding to healthcare expenses occurring between discharge and day 14 after exacerbation onset.

Maintenance costs were defined as non-exacerbation related cost after the exclusion of COPD drug costs [16].

Pneumonia costs were based on three diagnosis-related group (DRG) codes (D47A, D47C and D47E) describing lung inflammation with three levels of complications [23]. As no case mix information was available, an average of all three was assumed (Table 2). Costs were inflated to the year 2015 where necessary using the Harmonized Indices of Consumer Prices [24] and are expressed in 2015 euros (€) using European Central Bank foreign exchange reference rates (2015 annual average SEK/€ rate, 9.35:1 or 1:0.107) [25].

#### Utilities

Utilities, which represent the strength of a society’s preference for specific health-related outcomes, were calculated at the end of each cycle depending on disease severity status and other characteristics, based on a regression model by Rutten-van Mölken et al. [26] (see Additional file 1). The co-variate values were informed by the characteristics of simulated patients in the model. Baseline characteristics, including gender, body mass index etc. were derived from the FLAME trial baseline data [8]. FEV_1_% predicted over the time horizon of the model is described under disease progression. ER visits and hospitalisation admissions were linked to the incidence of moderate exacerbations and severe exacerbations, respectively, predicted by the model for each comparator.

Both costs and health benefits were discounted annually at the rate of 3% according to Swedish guidelines [27].

#### Mortality

Swedish life tables from Statistics Sweden for 2015 were used to generate background all-cause mortality [28]. Overall mortality was calculated by applying a COPD specific hazard ratio of 1.02 based on the Obstructive Lung Disease in Northern Sweden COPD study [29]. Supplementary details can be found in the Additional file 1. This hazard ratio was adjusted by the predicted decline in FEV_1_ for an individual patient, using the following equation:

Probability of death = (general population risk for the appropriate age and gender) * 1.02^ (the decline in FEV_1_ percent predicted).

Exacerbations themselves, in fact, did not affect mortality in this model. Both the rate of exacerbations and COPD-related mortality rate were based upon FEV_1_ status.

### Model outputs

The model outputs analysed in terms of health benefit were: life-years (LYs) and quality-adjusted life-years (QALYs). QALYs were calculated as a product of the quantity (LYs) and quality (utilities) of life lived. The model output in terms of cost was the total monetary cost which comprised drug, maintenance, exacerbation, and pneumonia costs. Net monetary benefit (NMB) was also estimated using the following formula [30]: NMB = (WTP*∆E) - ∆C, where WTP is the willingness to pay (per QALY) threshold, ΔE is the difference in effectiveness (e.g. number of QALYs) and ΔC is the difference in costs. NMB >0 would indicate that IND/GLY was cost-effective at the given WTP threshold.

Number needed to treat (NNT), which represents the average number of patients who need to be treated to prevent one patient from having an exacerbation was estimated for exacerbations. NNTs were calculated based on the following equation:

NNT = 1/ ((proportion benefiting from an intervention)-(proportion benefiting from a control)).

The NNT to prevent one severe exacerbation and one case of repeat exacerbation were also calculated and data are presented in the Additional file 1.

### Sensitivity analyses

Both one-way sensitivity analyses and probabilistic sensitivity analyses (PSA) were performed to acknowledge the uncertainty of key model input values and to test the robustness of the results.

One-way sensitivity analyses were conducted by changing each model input value discretely by 25%, keeping other model input values constant to identify key parameters affecting the results. The model inputs studied in the one-way sensitivity analyses were: exacerbation rate ratio, exacerbation severity, drug costs, FEV_1_ benefit, exacerbation costs, baseline rate of exacerbations, maintenance costs, pneumonia costs, and discount rates (0, 2.5, 3.5, 4, 5, 7, and 10%, for both costs and effects).

Details of the PSA can be found in the Additional file 1.

### Subgroup analyses

Subgroup analyses were also performed with respect to smoking (yes vs no), severity status (severity of airflow limitation (GOLD stages 1, 2, 3 and 4), and GOLD 2015-A, B, C and D categories based on symptoms, airflow limitation and risk of exacerbations) and gender (male vs female).

## Results

### Base case

IND/GLY was associated with lower costs and better outcomes compared with SFC over all the analysed time horizons of 1, 5, 10 years and lifetime. Compared with SFC, treatment with IND/GLY resulted in the addition of 0.192 LYs and 0.134 QALYs as well as cost savings of €1211 per patient over lifetime. Table 3 presents the results for the other time horizons (1, 5, and 10 years). As compared to the findings at 1 year, greater cost savings and more benefits were observed at the extended time horizons.

**Table 3:** Incremental results for the base case cost-effectiveness analysis for IND/GLY versus SFC

Outcomes	Time horizon (years)	IND/GLY	SFC	Incremental (IND/GLY-SFC)
QALYs (per patient)	1	0.617	0.615	0.002
	5	2.513	2.495	0.018
	10	4.102	4.054	0.047
	Lifetime	5.653	5.520	0.134
LYs (per patient)	1	0.979	0.979	0.000
	5	4.132	4.117	0.015
	10	6.780	6.726	0.055
	Lifetime	9.328	9.137	0.192
Total costs (€)(per patient)^a^	1	5406	5621	−214
	5	29,486	30,620	−1134
	10	50,062	51,716	−1654
	Lifetime	68,406	69,618	−1211

*IND/GLY* indacaterol/glycopyrronium, *LYs* life-years, *QALYs* quality-adjusted life-years, *SFC* salmeterol/fluticasone

^a^Negative numbers indicate cost savings (e.g., IND/GLY results in savings of €1654 per patient over a time horizon of 10 years compared to SFC)

Fewer moderate and severe exacerbations were reported with the use of IND/GLY compared with SFC over all the time horizons (Table 4). Since severe exacerbations are defined as those requiring hospitalization in addition to treatment with systemic corticosteroids and/or antibiotics, the results imply that hospitalization rates tend to be lower with IND/GLY compared with SFC.

**Table 4:** Number of exacerbations over the time horizons

Time horizon (years)	IND/GLY	SFC	Incremental
All exacerbations (moderate and severe)			
1 year	0.57	0.68	−0.11
5 years	3.26	3.92	−0.66
10 years	5.83	6.96	−1.13
Lifetime	8.62	10.09	−1.48
Moderate exacerbations			
1 year	0.47	0.57	−0.11
5 years	2.73	3.27	−0.55
10 years	4.87	5.81	−0.94
Lifetime	7.20	8.44	−1.24
Severe exacerbations			
1 year	0.09	0.11	−0.02
5 years	0.54	0.65	−0.11
10 years	0.96	1.15	−0.18
Lifetime	1.41	1.66	−0.24

Furthermore, the incidence of pneumonia was lower among patients receiving IND/GLY compared to those receiving SFC over the lifetime horizon (0.39 vs. 0.58).

The NMB was estimated to be €8560 based on a WTP threshold of €55,000/QALY.

The NNT to prevent one moderate or severe exacerbation was estimated to be 5 (4.76), i.e., for every ~5 patients treated over 12 months with IND/GLY rather than SFC, on average, one exacerbation was avoided. Or, in other words, if ~5 patients with moderate to very severe airflow limitation and ≥1 exacerbation in the preceding year are treated with IND/GLY instead of SFC, one exacerbation can be prevented.

### Uncertainty analyses

**Fig. 2:**
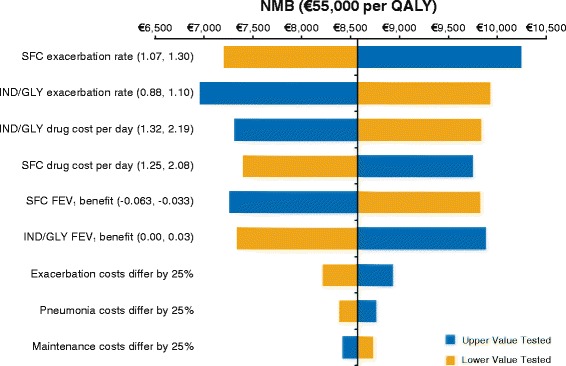
Net monetary benefit for the sensitivity analysis. Figure notes: FEV1: forced expiratory volume in 1 s; IND/GLY: indacaterol/glycopyrronium; NMB: net monetary benefit; QALY: quality-adjusted life-year

NMB was also positive after variation in the values of the following parameters: SFC exacerbation rate (1.07, 1.30), SFC cost per day (1.25, 2.08), IND/GLY FEV_1_ benefit (0.00, 0.03), exacerbation costs (25% variation) and pneumonia costs (25% variation) (Fig. 2) and when different discount rates were applied (data not shown). This indicates IND/GLY continues to be cost-effective under each of these alternative scenarios.

Results for the PSA are presented in the Additional file 1.

### Subgroup analyses

**Fig. 3:**
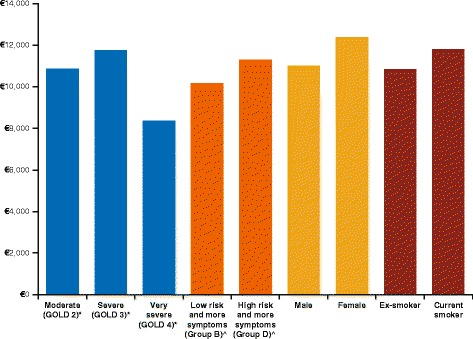
Net monetary benefit analysis for subgroups. Figure notes: *Severity of airflow limitation based on 2011–2014 GOLD criteria; ^Based on 2015 GOLD staging system. GOLD: global initiative for chronic obstructive lung disease classification

At a WTP threshold of €55,000/QALY and considering the variables- severity, gender and smoking status, the NMB increased further in the following subgroups: severe (GOLD 3, 2011–2014 criteria), high risk and more symptoms (GOLD D, 2015 criteria), female gender, and current smokers (Fig. 3).

## Discussion

Results of this analysis indicated that IND/GLY is cost saving with respect to reduction in exacerbation and pneumonia costs, and associated with favourable clinical effects such as reduced rate of moderate and severe exacerbations and lower incidence of pneumonia compared with SFC. These results were robust in uncertainty analyses. With various time horizons assessed, both immediate and lifelong benefits were observed with IND/GLY over SFC implying the beneficial use of IND/GLY for short-term or long-term policy planning. To highlight, the decrease in cost savings from 10 years to lifetime was due to increasing maintenance costs. At 10 years, IND/GLY is delaying transition to GOLD state IV and therefore is saving cost, but by lifetime almost all patients (who don’t die of comorbidities or other reasons) progress to GOLD IV so the cumulative savings in maintenance costs is less pronounced. Hence, greater drug costs for IND/GLY over this more distal time period results in overall costs being slightly higher for IND/GLY compared to SFC. Results from the pre-specified subgroup analyses also suggest that even in much targeted use, IND/GLY is the preferable treatment option compared to SFC.

Results of this study are in line with the previously conducted CEA in the Swedish setting, which suggested that IND/GLY is associated with cost savings and is more effective than SFC in moderate to severe COPD patients with no history of exacerbations [13]. Recent studies have also demonstrated cost-effectiveness of dual bronchodilators over monotherapies in patients with COPD [31, 32].

To the best of our knowledge, this is the first study to assess the cost-effectiveness of IND/GLY versus SFC utilising clinical data from the FLAME trial, which assessed COPD patients with moderate to very severe airflow limitation and ≥1 exacerbation in the preceding year. Results from the present analyses become more relevant with the recent recommendations from GOLD suggesting the first-line option of dual bronchodilators in many symptomatic COPD patients, regardless of their exacerbation risk. The subgroup analyses highlight specific populations in which IND/GLY can be an economic advantage over the use of SFC in settings with restrained budgets. The current analysis not only focused on the cost-effectiveness outcomes, but also on outcomes relevant for clinical practice such as exacerbations, pneumonia and NNT.

There are also several limitations to this study. Results from this analysis are relevant to patients resembling the studied population in the FLAME trial i.e. COPD patients with mMRC dyspnea grade ≥ 2, moderate to very severe airflow limitation and ≥1 exacerbation in the preceding year. We can only speculate that inhaler technique and lower adherence in real-life patients as compared to a clinical trial population would influence treatment effectiveness in a similar way for all maintenance treatments, hence not affecting differences between them; however, we acknowledge the lack of data on this specific point in the literature. The modelling approach used is largely in line with previously published models in COPD. However, despite recent recommendations, costs related to comorbidities and some adverse effects could not be included for IND/GLY and SFC separately due to lack of corresponding data [15]. While the most significant and relevant adverse events (exacerbations and pneumonia) were included in the model as reported in the FLAME study [8], other adverse events were omitted as the rates were small and similar between the two treatment groups while corresponding costs are low. Thus their inclusion would not meaningfully inform the cost-effectiveness analysis. Lastly, the study selected the Swedish implicit threshold of €55,000 (dependent on the unmet need and severity of the disease), for which no official reference is available.

Since the applicability of findings from a controlled environment to real-world settings is uncertain hence future research is recommended to follow-up clinical and economic implications of these findings in real life.

In patients with moderate to very severe COPD, LABA/ICS fixed-dose combination is known to reduce the frequency of COPD exacerbations [33, 34], and it is also no doubt that some patients may actually benefit from the addition of ICS. However, an indiscriminate and long-term use of ICS in these patients may expose them to an increased risk of developing pneumonia leading to increased associated healthcare costs [35]. Therefore, as recommended by GOLD, most patients with moderate to very severe airflow limitation and a history of exacerbations should be treated with LABA/LAMA combination before using LABA/ICS.

Until now, the cost-effectiveness of LABA/LAMA combination vs. LABA/ICS combination in COPD population with moderate to very severe airflow limitation and ≥1 exacerbation in the preceding year was uncertain. This study provides an answer to this so far unaddressed question, showing that from a health economic perspective, IND/GLY is not only cost-effective but should be the preferred treatment option compared to SFC in COPD patients with moderate to very severe airflow limitation and ≥1 exacerbation in the preceding year.

## Conclusion

Under the current WTP threshold in Sweden, IND/GLY is a cost-effective treatment compared with SFC in COPD patients with mMRC dyspnea grade ≥ 2, moderate to very severe airflow limitation, and a history of exacerbations. Cost savings were observed with respect to reduction in exacerbation and pneumonia costs, and superior efficacy in terms of reduced rate of moderate and severe exacerbations and lower incidence of pneumonia. Sensitivity analyses performed considering key parameters also confirm the results for their robustness.

## Additional file

Additional file 1: Methods and Results (additional details) (ZIP 535 kb)

## Abbreviations

CEAs: cost-effectiveness analyses
COPD: chronic obstructive pulmonary disease
DRG: diagnosis-related group
FEV1: forced expiratory volume in 1 s
FVC: forced vital capacity
ICS: inhaled corticosteroid
IND/GLY: indacaterol/glycopyrronium
LABA: long-acting β2-adrenergic agonist
LAMA: long-acting muscarinic antagonist
mMRC: modified Medical Research Council
NMB: net monetary benefit
NNT: Number needed to treat
PSA: probabilistic sensitivity analyses
QALYs: quality-adjusted-life-years
SEK: Swedish krona
SFC: salmeterol/fluticasone
