# Supplementary material for: Indacaterol/glycopyrronium is cost-effective compared to salmeterol/fluticasone in COPD: FLAME-based modelling in a Swedish population
Source: Respir Res. 2017 Dec 11;18:206. doi: 10.1186/s12931-017-0688-5 (PMC5725803; doi:10.1186/s12931-017-0688-5)
Supplement: Additional file 1: — Methods and Results (additional details) (ZIP 535 kb) [file 12931_2017_688_MOESM1_ESM.zip › Additional file 1.DOCX]

**Methods (additional details)**

**Exacerbations**

In the FLAME trial, a COPD exacerbation was defined as worsening of the following two or more major symptoms for ≥2 consecutive days: dyspnoea, sputum volume, and sputum purulence OR worsening of any one major symptom together with an increase in any one of the following minor symptoms for ≥2 consecutive days: sore throat, cold, fever without other cause, cough, and wheezing [1]. The symptoms were collected through daily electronic diaries and at study visits. The type of treatment given for a COPD exacerbation determined its severity [1]:

- Moderate: A worsening of symptoms that met the above symptom definition that was treated with systemic corticosteroids or antibiotics or both.
- Severe: Requiring hospitalisation (>24 hours emergency room visit) in addition to treatment with systemic corticosteroids and/or antibiotics.

**Pneumonia**

In FLAME trial, pneumonia was defined as an event characterised by increased respiratory symptoms: increased cough, dyspnoea, wheezing, purulent sputum and fever (body temperature >38°C) or pleuritic chest pain or leukocytosis or other clinical signs consistent with pneumonia considered relevant in the opinion of the investigator [1]. Radiographic imaging (chest X-ray or computed tomography) was required to confirm the diagnosis [1].

**Costs**

In the ARCTIC study, exacerbation costs were defined according to exacerbation severity. Moderate exacerbations were defined as visits to primary care for COPD (ICD-10: J44) and/or collection of oral steroids (ATC H02AB) or antibiotics targeted at respiratory diseases (ATC J01AA/J01CA) [2]. Severe exacerbations were defined as hospitalisation due to COPD exacerbation in secondary care (ICD-10: J44.1) and/or emergency department visits (J44.1). Recurrent exacerbations occurring within 14 days were considered as one unique event [2].

Table S1 shows the cost inputs taken from the ARCTIC study (in Swedish Krona, SEK). These were then inflated to 2015 costs for use in the present analysis.

**Table S1. ARCTIC cost inputs (SEK, 2013)**

| **Parameter** | **Mean estimate** | **Standard deviation** |
| --- | --- | --- |
| Moderate exacerbation cost per occurrence  Moderate airflow limitation (GOLD 2010 classification) | 5046 (median: 1828) | 8279 |
| Moderate exacerbation cost per occurrence  Severe airflow limitation (GOLD 2010 classification) | 4909 (median: 2049) | 6602 |
| Moderate exacerbation cost per occurrence  Very severe airflow limitation (GOLD 2010 classification) | 4458 (median: 2033) | 6539 |
| Severe exacerbation cost per occurrence  Moderate airflow limitation (GOLD 2010 classification) | 47901 (median: 33518) | 48960 |
| Severe exacerbation cost per occurrence  Severe airflow limitation (GOLD 2010 classification) | 47937 (median: 36695) | 47608 |
| Severe exacerbation cost per occurrence  Very severe airflow limitation (GOLD 2010 classification) | 66557 (median: 42493) | 71426 |
| Annual non-exacerbation related maintenance costs  Moderate airflow limitation (GOLD 2010 classification) | 55019 | - |
| Annual non-exacerbation related maintenance costs  Severe airflow limitation (GOLD 2010 classification) | 53390 | - |
| Annual non-exacerbation related maintenance costs  Very severe airflow limitation (GOLD 2010 classification) | 60189 | - |

**Utilities**

The following regression equation was used to calculate the utility value for each patient at each cycle:

Utility value = 0.688 + Gender*0.057 + FEV1 percent predicted* 0.003 + Emergency visits in the last year*-0.029 + Hospital admission in the last year * -0.02 + Number of concomitant diseases*-0.01 + BMI*-0.003

**Mortality**

The Obstructive Lung Disease in Northern Sweden COPD study evaluated the impact of COPD on mortality and reported that FEV1 at baseline was an independent significant risk factor for death with hazard ratio reported to be 0.98 [3]. This value indicated that for every one unit (percentage) increase in FEV1 percent predicted, the risk of death was 0.98 of the reference risk. In the present analysis, since FEV1 showed a decline over the course of the model, the inverse of the hazard ratio was used (1/0.98) such that the risk of death was 1.02 of the reference risk of death for every one unit decline in FEV_1_ percent predicted [3].

**Results (Additional details)**

**NNT to prevent one severe exacerbation**

The NNT to prevent one severe exacerbation, where a severe exacerbation is defined as a COPD exacerbation requiring hospitalization, was estimated to be 50, i.e. for every 50 people treated over 12 months with IND/GLY rather than SFC, on average, one severe exacerbation will be avoided.

**NNT to prevent one case of repeat exacerbation**

The NNT to prevent one case of repeat exacerbations, where repeat exacerbations is defined as patients presenting with 3 or more COPD exacerbations over 12 month trial period, was estimated to be 32 (31.58), i.e. for every 32 people treated over 12 months with IND/GLY rather than SFC, on average, one fewer will experience 3 or more exacerbations.

**Probabilistic sensitivity analysis**

A probabilistic sensitivity analysis (PSA) was performed by sampling from 10,000 patients and 1000 cohorts, based on Monte Carlo methodology, where each cohort represented an alternative set of model input values drawn from appropriate distributions.

Table S2 and Table S3 provide distribution for efficacy inputs and other model inputs, respectively.

**Table S2. Probabilistic sensitivity analysis distribution for efficacy inputs**

| **Parameter** | **PSA distribution** |
| --- | --- |
| Annual rate of moderate and severe exacerbations | Log-normal |
| Improvement in pre-dose trough FEV1 from baseline in liters at 52 weeks | Normal |
| Pneumonia | Log-normal |

PSA: Probabilistic sensitivity analysis

**Table S3. Distributions used for other model inputs**

| **Parameter** | **Distribution** |
| --- | --- |
| **Moderate exacerbation cost per occurrence** |  |
| Mild | Gamma |
| Moderate | Gamma |
| Severe | Gamma |
| Very severe | Gamma |
| **Severe exacerbation cost per occurrence** |  |
| Mild | Gamma |
| Moderate | Gamma |
| Severe | Gamma |
| Very severe | Gamma |
| **Annual non-exacerbation related maintenance costs** |  |
| Mild | Gamma |
| Moderate | Gamma |
| Severe | Gamma |
| Very severe | Gamma |
| **Pneumonia event** |  |
| Cost per occurrence | Gamma |
| **Clinical Parameter's** |  |
| Annual rate of moderate and severe exacerbations | Log Normal |
| Improvement in pre-dose trough FEV1 from baseline in liters at 52 weeks | Normal |
| Pneumonia | Log Normal |
| **Utility** |  |
| Constant | Normal |
| Gender | Normal |
| FEV1 percent predicted | Normal |
| Emergency visits in the last year | Normal |
| Hospital admissions in the last year | Normal |
| Number of concomitant disease | Normal |
| **Mortality** |  |
| Hazard Ratio | Log Normal |
| **Baseline characteristics** |  |
| Age at baseline, mean (SD), years | N/A |
| Height, mean (SD), cm | N/A |
| Weight, mean (SD), kg | N/A |
| BMI, mean (SD), kg/m^2^ | N/A |
| Proportion males (%) | N/A |
| **Severity of COPD** |  |
| GOLD 1 | N/A |
| GOLD 2 | N/A |
| GOLD 3 | N/A |
| GOLD 4 | N/A |
| Group A | N/A |
| Group B | N/A |
| Group C | N/A |
| Group D | N/A |
| Number of COPD exacerbations in the previous year | N/A |
| Current smokers | N/A |

BMI: Body Mass Index; FEV1: Forced Expiratory Volume in 1 second; GOLD: Global Initiative for Chronic Obstructive Lung Disease; SD: Standard Deviation

The PSA scatter plot shows simulation iterations plotted for incremental costs and incremental QALYs indicating the level of certainty in the base case analysis. The scatter plot shows that IND/GLY was dominant over SFC, in 66% of the simulations at a threshold of €0, and in 100% of the simulations at a threshold of €20000, respectively (Figure S1).

**Figure S1. Probabilistic sensitivity analysis scatter plot of indacaterol/glycopyrronium versus salmeterol/fluticasone**


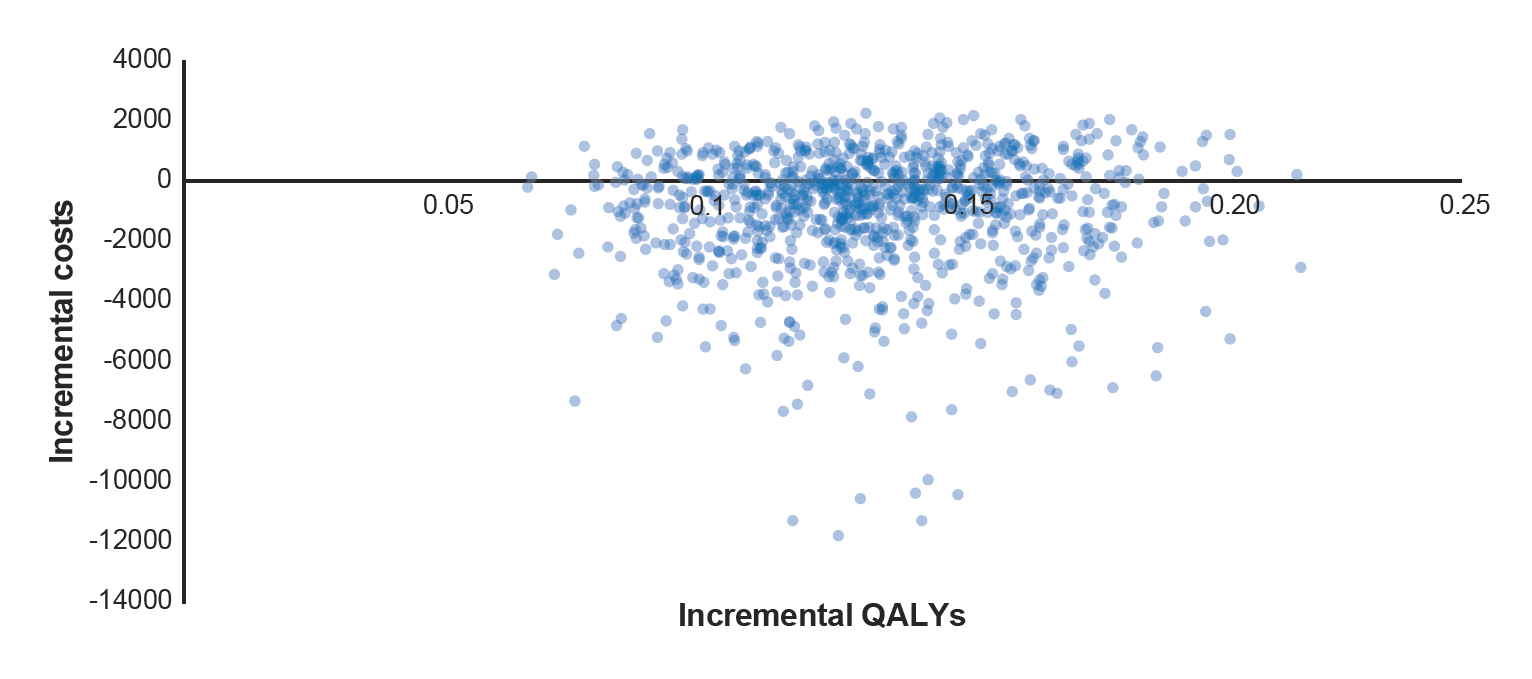
 QALYs Quality-Adjusted Life-Years

A cost-effectiveness acceptability curve was generated from the results of the PSA. This curve indicates the probability of a treatment being cost-effective compared to the other treatment at a given ICER threshold of €55000 roughly equivalent to 500000 SEK based on 2015 average exchange rate (Figure S2) [4]. This ICER threshold is also known as the willingness-to-pay (WTP) threshold, which signifies the maximum amount a payer is willing to pay per QALY gained.

**Figure S2. Cost-effectiveness acceptability curve of indacaterol/glycopyrronium versus salmeterol/fluticasone**


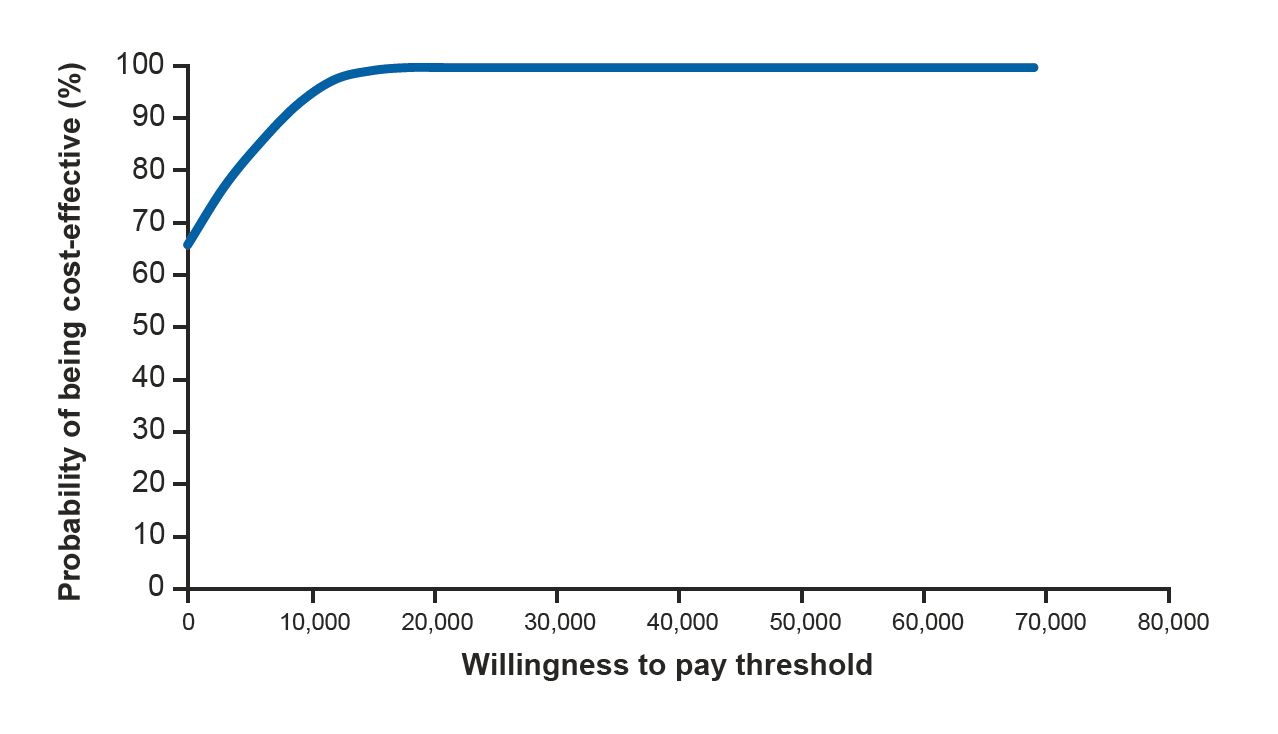


**References**

1. Wedzicha JA, Banerji D, Chapman KR, Vestbo J, Roche N, Ayers RT, Thach C, Fogel R, Patalano F, Vogelmeier CF, Investigators F: Indacaterol-Glycopyrronium versus Salmeterol-Fluticasone for COPD. N Engl J Med 2016, 374:2222-2234.

2. The economic burden of COPD in a Swedish cohort: The ARCTIC study. 2016. <http://www.nature.com/article-assets/npg/npjpcrm/abstracts/npjpcrm201622.pdf>. Accessed August 03, 2016.

3. Lindberg A, Larsson LG, Muellerova H, Ronmark E, Lundback B: Up-to-date on mortality in COPD - report from the OLIN COPD study. BMC Pulm Med 2012, 12:1.

4. Nationella riktlinjer för sjukdomsförebyggande metoder, Hälsoekonomiskt underlag (bilaga). Stockholm, Socialstyrelsen. 2011. <http://www.socialstyrelsen.se/nationellariktlinjerforsjukdomsforebyggandemetoder/Documents/nr-sjukdomsforebyggande-halsoekonomisktunderlag.Pdf>. Accessed November 17, 2016.
