# Supplementary figures and images for: Indacaterol/glycopyrronium is cost-effective compared to salmeterol/fluticasone in COPD: FLAME-based modelling in a Swedish population
Source: Respir Res. 2017 Dec 11;18:206. doi: 10.1186/s12931-017-0688-5 (PMC5725803; doi:10.1186/s12931-017-0688-5)

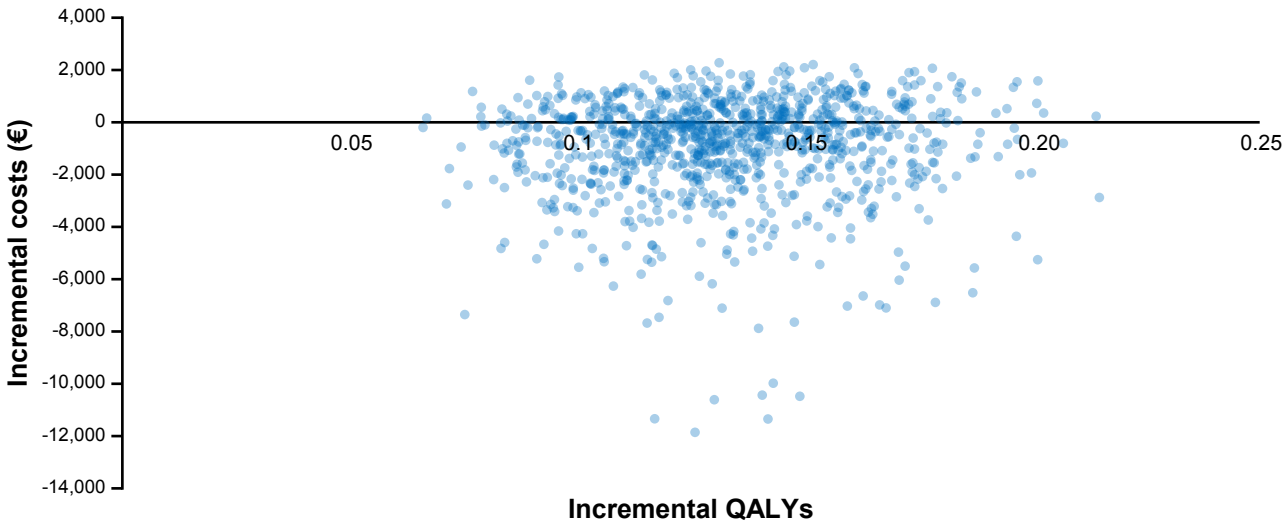

Supplement: Additional file 1: — Methods and Results (additional details) (ZIP 535 kb) [file 12931_2017_688_MOESM1_ESM.zip › Fig S1.pdf]

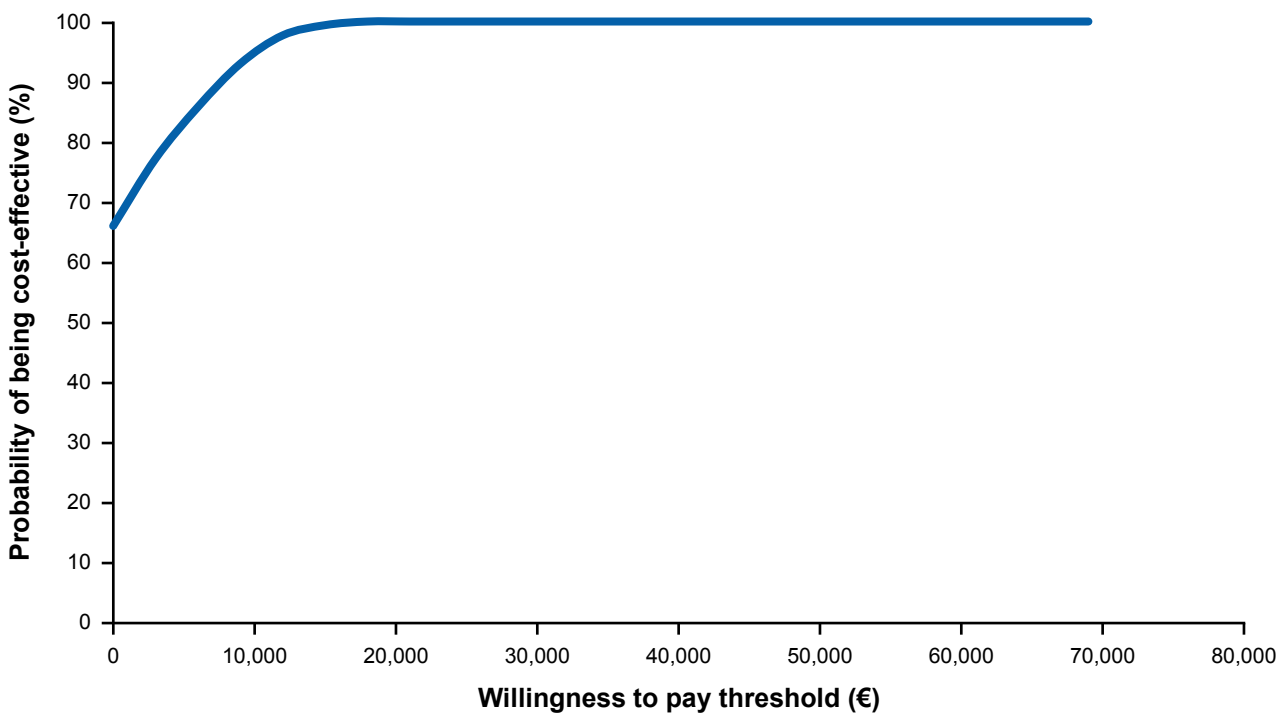

Supplement: Additional file 1: — Methods and Results (additional details) (ZIP 535 kb) [file 12931_2017_688_MOESM1_ESM.zip › Fig S2.pdf]
